# An 85-amino-acid polypeptide from *Myrmeleon bore* larvae (antlions) homologous to heat shock factor binding protein 1 with antiproliferative activity against MG-63 osteosarcoma cells in vitro

**DOI:** 10.2478/abm-2022-0024

**Published:** 2022-08-31

**Authors:** Rui Ding, Ming He, Huoying Huang, Jing Chen, Mingxing Huang, Yonghui Su

**Affiliations:** Department of General Surgery, The Fifth Affiliated Hospital, Sun Yat-Sen University, Zhuhai, Guangdong 519000, China; Department of Biological Engineering, School of Biomedical and Pharmaceutical Science, Guangdong University of Technology, Guangzhou, Guangdong 510006, China

**Keywords:** antiproliferative agent, arthropod venoms, HSBP1 protein, osteosarcoma, polypeptide

## Abstract

**Background:**

Venomous arthropods have substances in their venom with antiproliferative potential for neoplastic cells.

**Objectives:**

To identify a polypeptide from *Myrmeleon bore* (antlion) with antiproliferative activity against neoplastic cells, and to elucidate the molecular mechanism of the activity.

**Methods:**

We used gel filtration and ion exchange chromatography to purify a polypeptide with antiproliferative activity against MG-63 human osteosarcoma cells from a proteinaceous extract of antlion. The polypeptide was sequenced and the stability of its antiproliferative activity was tested under a range of conditions in vitro. An 3-(4,5-dimethylthiazol-2-yl)-2,5-diphenyltetrazolium bromide (MTT) assay was used to determine the antiproliferative activity of the polypeptide against the MG-63 osteosarcoma cells and MC3T3-E1 mouse calvarial osteoblasts, which were used as a non-neoplastic control. We used western blotting to compare the levels of expression of heat shock transcription factor 1 (HSF1), heat shock protein 90 (HSP90), cyclin-dependent kinase 4 (CDK4), and protein kinase B alpha (ATK1) in MG-63 osteosarcoma cells and their mouse homologs in MC3T3-E1 osteoblasts after their treatment with the antlion antiproliferative polypeptide (ALAPP).

**Results:**

The 85-amino-acid ALAPP has a 56% sequence identity with the human heat shock factor binding protein 1 (HSBP1). The antiproliferative activity of the polypeptide is relatively insensitive to temperature, pH, and metal ions. ALAPP has a strong concentration-dependent antiproliferative activity against MG-63 osteosarcoma cells compared with its effect on MC3T3-E1 osteoblasts. ALAPP significantly upregulates the expression of HSF1 in MC3T3-EL osteoblasts, but not in MG-63 osteosarcoma. ALAPP significantly downregulated the expression of HSP90, CDK4, and AKT1 expression in MG-63 osteosarcoma, but not in the osteoblasts.

**Conclusions:**

ALAPP has significant antiproliferative activity against MG-63 osteosarcoma cells, but not nonneoplastic MC3T3-E1 osteoblasts. We speculate that non-neoplastic cells may evade the antiproliferative effect of ALAPP by upregulating HSF1 to maintain their HSP90, CDK4, and AKT1 expression at a relatively constant level.

Polypeptides as therapeutic substances are regarded as being highly specific, efficacious, relatively safe, and well tolerated, and as a result there is growing interest in their therapeutic use in a wide range of disease areas such as antibiotic-resistant bacteria, cancer, and metabolic disorders [1]. Polypeptide agents with potential antiproliferative activity are often small, with a cationic amphipathic structure that enables them to interact with anionic constituents on lipid membranes [2, 3]. The substances available from natural sources as the quintessence of natural evolutionary programs seem to be unlimited, and the value of natural products as sources of new drugs is highlighted by the large proportion of drugs in clinical use having a natural product origin. Nature produces an impressive myriad variety of biologically active peptides, and, therefore, presents one of the most promising sources for peptide drug discovery. Animals are a treasure trove of bioactive peptides that have evolved high affinity and selectivity for a diverse range of biological targets. Among the animals that produce pharmacologically active molecules are arthropods, such as scorpions, bees, wasps, spiders, and ants, whose venoms have been found to contain substances with great antiproliferative potential.

Antlions are the larvae of the *Myrmeleon bore* from the superfamily Myrmeleontoidea, belonging to the order Neuroptera, and family Myrmeleontidae, which has a metamorphic developmental lifecycle [4, 5]. In traditional Chinese medicine (TCM), antlions are used to treat a variety of diseases, such as renal calculi and stranguria, trauma, and furuncles resulting from trauma [6]. Medical studies of TCM have shown that extracts of antlions can significantly alleviate thrombosis and osteomyelitis. Preliminary pharmacological and toxicological experiments showed that the alcohol or water extracts of antlions could have analgesic, central excitatory, detumescent (anti-inflammatory) effects, and inhibit thrombus formation in rats [7]; and decrease inflammation [8] and enhance nonspecific and humoral immunity in mice [9]. In TCM, some formulae containing antlion (also known as “Jinsha ox”) are recommended for treating tumors [10, 11]. In the present study, we investigated the antiproliferative activity of a proteinaceous antlion extract and its chromatographically fractionated components to identify proteins and peptides in the extract with antiproliferative activity against a neoplastic cell line. We then characterized the purified polypeptide we had isolated to determine its stability and elucidate the possible molecular mechanism of its antiproliferative activity.

## Methods

The present study included only *Myrmeleon bore* insect larvae (antlions) and commercial cell lines in vitro and was therefore exempted from requirements for Institutional Review Board or animal ethics review.

### Antlion extract preparation and electrophoresis

Antlions of about 3–4 mm in length were collected in Zhanjiang, Guangdong province, and washed clean with distilled water. The larvae were divided into 10 g aliquots and preserved at −80 °C. Antlions (about 250–300 larvae) were ground with liquid nitrogen and homogenized in 250 mL of 50 mM sodium phosphate buffer (pH 7.4) with 0.15 M NaCl. After leaching overnight at 4 °C, the mixture was centrifuged at 15,000 ×*g* for 10 min ×2 times at 4 °C to pellet out the cell debris, and lipids were removed by filtration through qualitative filter paper at medium speed to obtain a proteinaceous extract.

Protein concentration was determined by the method of Bradford [12]. Bovine serum albumin (BSA, 1 mg/mL) solution was used as standard. The absorbance of the dye–protein reaction mixtures in the wells of 96-well plates was measured at 570 nm using a microplate reading spectrophotometer (iMark, Bio-Rad).

The protein and polypeptide components of the antlion extract and purified fractions were separated, and the molecular weights of the components were determined by modified Tricine–sodium dodecyl (SDS)–polyacrylamide gel electrophoresis (PAGE) using a 14% gel (3.3% crosslinking) with 0.1% SDS in the electrode and gel buffers [13, 14]. Coomassie Brilliant Blue R-250 (0.1% w/v) was used to stain the protein bands. We used a LowRange Protein molecular weight marker set (catalog No. C600201; Sangon Biotech, Shanghai, China).

### Gel filtration chromatography

Sephacryl-S100-HR (GE Healthcare) was packed into a column (16 mm × 60 mm), which was equilibrated with 2 column volumes of 50 mM Tris-HCl buffer (pH 7.4) containing 0.15 M NaCl at 1 mL/min. After concentration by ultrafiltration using a Microsep centrifugal device (3 kDa molecular weight cut-off, Pall Corporation), 1 mL of the proteinaceous antlion extract was loaded onto the top of the gel. The column was eluted with the equilibration buffer at 0.5 mL/min, and the eluent was collected into 2 mL aliquots. The antiproliferative activity of each fraction against MG-63 human osteosarcoma cells was measured using an 3-(4,5-dimethylthiazol-2-yl)-2,5-diphenyltetrazolium bromide (MTT) cell viability assay.

### Ion exchange chromatography

Q-Sepharose-FF (GE Healthcare) was packed into a column (10 mm × 100 mm), which was equilibrated with 5 column volumes of 50 mM Tris-HCl (pH 7.6) at 5 mL/min. After gel filtration, fractions enriched in low-molecular-weight antiproliferative active polypeptide component were desalted against 50 mM Tris-HCl (pH 7.6) by ultrafiltration using a Microsep device (3kDa molecular weight cut-off), and the mixture loaded onto the ion exchange column at 2 mL/min. The column containing the loaded polypeptide mixture was then eluted with about 5 column volumes of equilibration buffer until absorbance had reached baseline, and the bound proteins were then eluted with 5 column volumes of 50 mM Tris-HCl (pH 7.6) containing a stepwise gradient of NaCl (0–0.5 M).

### Cell culture and antiproliferative viability assay

The human osteosarcoma cell line MG-63 (ATCC CRL-1427) was cultured in Eagle's Minimum Essential Medium (MEM), and the mouse osteoblast cell line MC3T3-E1 subclone 14 derived from osteoblast precursors of the parietal calvaria (ATCC CRL-2594) was cultured in alpha Minimum Essential Medium (α-MEM). Both media were supplemented with 10% fetal bovine serum, and the cells were incubated at 37 °C under an atmosphere of 5% CO_2_. Both cell lines were subcultured every 4–5 d after reaching 85%–95% confluence with medium renewal every 2–3 d. The cell cultures were trypsinized and adjusted to 5 × 10^4^ cells/mL. Cell suspensions were seeded in 96-well plates at 5 × 10^3^ cells per well (100 μL/well) and cultured overnight until cells had adhered to the well wall. The cells in the 96-well plate were treated for 48 h with purified antlion antiproliferative polypeptide (ALAPP) in a gradient of concentrations or with phosphate-buffered saline (PBS) as control. The morphological characteristics and changes in cells were evaluated using an inverted phase contrast microscope EVOS FL Auto Cell Imaging System fitted with a 20×LPlan FH objective (catalog No. AMEP-4682; Thermo Fisher Scientific) in monochrome transmission mode.

Cell viability and, therefore, antiproliferative activity was determined using an 3-(4,5-dimethylthiazol-2-yl)-2,5-diphenyltetrazolium bromide (MTT) assay according to the conversion of pale yellow MTT tetrazole to purple formazan crystals by viable cells [15]. The MTT reagent (10 μL) was added to each well of a 96-well plate containing cell culture and incubated for 4 h at 37 °C. After incubation, 100 μL of MTT solubilization buffer (equal to the volume of the original culture medium) was added to each well to dissolve the formazan crystals. Within 1 h, the absorbance of the well contents was measured at 490 nm on a microplate reading spectrophotometer (iMark).

### Effect of pH and temperature on ALAPP stability

To determine the polypeptide stability at various pH, 50 mM acetate buffer (for pH 2–5.5), 50 mM Tris-HCl buffer (for pH 6–8.5), and 20 mM sodium carbonate-NaOH buffer (for pH 9–11) were used for a pH gradient (with an interval of 0.5 pH units). The ALAPP was incubated at each pH for 60 min at 4 °C. The pH was adjusted to 7.2 before measuring the antiproliferative activity of the treated peptide against MG-63 osteosarcoma cells using an MTT assay. The effect of temperature on polypeptide stability was tested after incubation at 20–70 °C in a water bath for 60 min followed by cooling in an ice bath. The antiproliferative activity of the heat-treated ALAPP against MG-63 osteosarcoma cells was then measured using an MTT assay.

### Effect of metal ions

Metal ions were added to the solution of ALAPP in 50 mM Tris-HCl (pH 7.6) at final concentrations varying from 0 to 30 mM to test their effect on the antiproliferative activity of the peptide against MG-63 osteosarcoma cells. The metal ions tested included Na^+^, K^+^, Mg^2+^, and Ca^2+^. Mixtures of ALAPP and the individual types and concentrations of metal ions were incubated at 25 °C for 30 min, and the relative antiproliferative activity of the mixtures was measured using an MTT assay against a control without any metal ions.

### Western blotting

About 5 mg of cell pellet was collected and mixed with 100 μL xTractor Buffer (Clontech). The crude lysate was centrifuged at 12,000 × *g* for 20 min at 4 °C to remove cell debris. All crude extracts were adjusted to the same protein concentration using the Bradford method, and protein components were separated by electrophoresis in a 12% polyacrylamide gel containing SDS and transferred to polyvinylidene difluoride (PVDF) membranes. Primary antibodies, including rabbit polyclonal antibodies to β-actin (anti-ACTC1, catalog No. D224905), heat shock transcription factor 1 (anti-HSF1, catalog No. D220782), heat shock protein 90 kDa alpha (cytosolic), class A member 1 (anti-HSP90AA1, catalog No. D220009), cyclin-dependent kinase 4 (anti-CDK4, catalog No. D120396), and serine-threonine protein kinase encoded by *AKT1* (anti-AKT1(Ab-129), catalog No. D151616), each with immunoreactivity for human and mouse homologs of the proteins, and secondary antibody of horseradish peroxidase (HRP)-conjugated goat anti-rabbit IgG (catalog No. D110058), were purchased from Sangon Biotech. The ECL chemiluminescence HRP substrate for western blotting (catalog No. T7101A) was purchased from TaKaRa and used in accordance with the protocols specified by the manufacturer. The expression of the various proteins was calculated relative to β-actin.

### Statistical analysis

All data are presented as mean and standard deviation. Assays were conducted in triplicate. Significant difference at the level of *P* < 0.05 after a Student *t* test was used to compare the values of parameters tested.

## Results

### Purification of ALAPP

**Figure 1:**
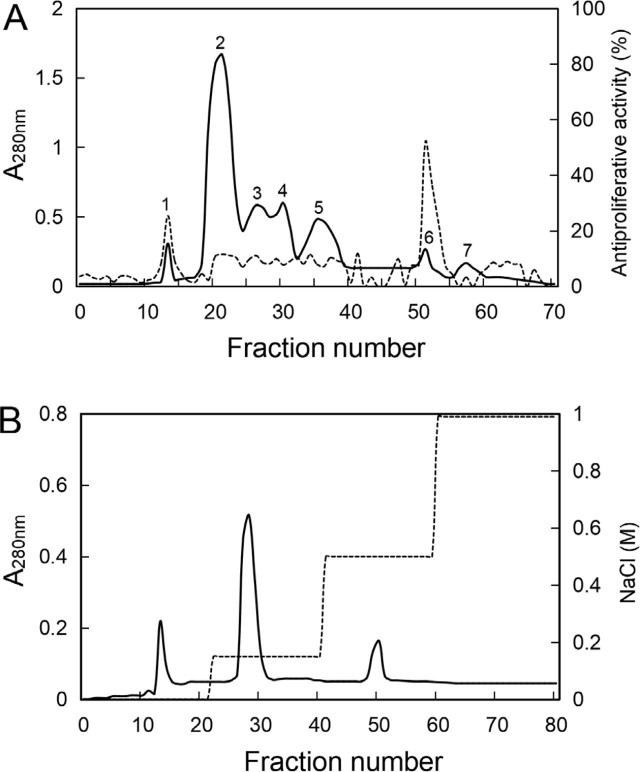
Purification of ALAPP by gel filtration and ion exchange chromatography. **(A)** Gel filtration in Sephacryl-S100-HR. The protein concentration in the eluate was estimated from its absorbance at 280 nm (solid line). Antiproliferative activity against MG-63 osteosarcoma cells (dashed line) was measured by MTT cell viability assay. Peak 1 (Fraction 13), Peak 2 (Fraction 20–22), Peak 3 (Fraction 26–27), Peak 4 (Fraction 29–30), Peak 5 (Fraction 35–37), Peak 6 (Fraction 50–51), and Peak 7 (Fraction 56–58). **(B)** Ion exchange chromatography on Q-Sepharose-FF. After elution with about 5 column volumes of equilibration buffer (50 mM Tris-HCl, pH 7.4) until absorbance reached baseline, bound proteins were eluted with equilibration buffer containing a stepwise gradient of NaCl (0–0.5 M) (dashed line), and the polypeptide with antiproliferative activity (ALAPP) was in the fraction eluted with the equilibration buffer containing 0.15 M NaCl. ALAPP, antlion antiproliferative polypeptide.

About 232 mL of a crude proteinaceous extract of antlions with a concentration of 5.2 mg/mL was obtained (about 12% total yield protein). The crude protein extract was concentrated by ultrafiltration and was applied to a Sephacryl-S100-HR column eluted with 50 mM Tris-HCl buffer (pH 7.4) containing 0.15 M NaCl. The gel filtration resulted in the separation of 7 protein peaks (**Figure 1A**). The corresponding relationship between the 7 peaks and the number of the fractions was as follows: Peak 1 (Fraction 13), Peak 2 (Fractions 20–22), Peak 3 (Fractions 26–27), Peak 4 (Fractions 29–30), Peak 5 (Fractions 35–37), Peak 6 (Fractions 50–51), and Peak 7 (Fractions 56–58). Antiproliferative activity against MG-63 osteosarcoma cells was mainly detected in Peak 1 and Peak 6. Peak 1, which mainly consisted of proteins with >100 kDa molecular weight, was not considered for further study due to the low stability and poor cell permeability of these proteins compared with smaller polypeptides. Only the 2 aliquots of the summit of Peak 6 with a total of 4 mL were selected for further study. The pooled fractions with antiproliferative activity from Peak 6 of the gel filtration chromatography were concentrated and the buffer was desalted against 50 mM Tris-HCl (pH 7.6) using ultrafiltration. The Peak 6 fraction pool in the new low-salt buffer was applied to a column of Q-Sepharose-FF. A polypeptide with antiproliferative activity against MG-63 osteosarcoma cells was eluted with 50 mM Tris-HCl (pH 7.6) containing 0.15 M NaCl and named antlion antiproliferative polypeptide (ALAPP) (**Figure 1B**).

**Figure 2:**
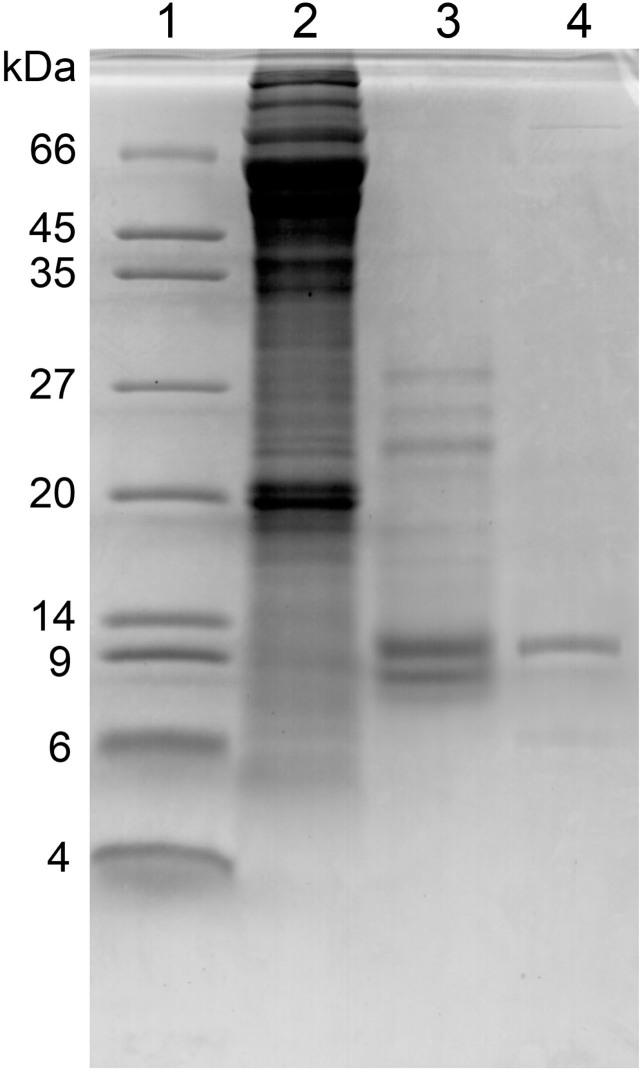
Purification profiles of ALAPP from antlion. Proteins in samples following each purification step were separated by Tricine–SDS–PAGE (14% polyacrylamide, 3.3% crosslinked) and stained with Coomassie Brilliant Blue R. Lane 1, protein molecular weight markers (catalog No. C600201; Sangon Biotech). Lane 2, crude protein extract. Lane 3, proteins of Peak 6 pool from Sephacryl-S100-HR. Lane 4, proteins eluted from Q-Sepharose-FF, including the ALAPP with antiproliferative activity. ALAPP, antlion antiproliferative peptide; PAGE, polyacrylamide gel electrophoresis; SDS, sodium dodecyl sulfate.

After the 3 steps of purification, a polypeptide with antiproliferative activity against MG-63 osteosarcoma cells (ALAPP) was purified from the crude antlion extract (**Table 1**). The final degree of purification was 60.5-fold with a total yield of 0.14%. The yield of each purification step was relatively low because the strategy employed was to obtain high purity, and not high recovery of the proteins, as its objective. The molecular weight of the ALAPP was estimated by Tricine–SDS–PAGE using a 14% polyacrylamide gel with 0.1% SDS in the electrode buffer and gel buffer. The relative molecular mass of ALAPP was estimated at 9.6 kDa (**Figure 2**). The band containing the ALAPP was excised from the gel and sent to Sangon Biotech for *N*-terminal Edman sequencing. The complete sequence obtained was:

MTDVKTTELNNEDVQNFTVSSSNDPKNMQELTQYVQTLLQTMQDKFQTMSDQIINRIDEMGNRIDDLEKNIADLMTQAGVEGPDK with a theoretical molecular weight of 9.714 kDa and pI 4.11 as calculated using the tool on the Expasy website (https://web.expasy.org/compute_pi/) operated by the SIB Swiss Institute of Bioinformatics [16].

**Table 1:** Purification of ALAPP from *Myrmeleon bore* larvae (antlions)

**Purification procedure**	**Total protein (mg)**	**IC_50_ (μg/mL)†**	**Purification degree (fold)**	**Yield (%)**
Crude protein extract	1206	1845	1.0	12
Gel filtration chromatography	60.2	102	18.2	5
Ion exchange chromatography	1.67	30.5	3.33	2.8

† Against MG-63 osteosarcoma cells.

ALAPP, antlion antiproliferative polypeptide.

### Biochemical properties of ALAPP

**Figure 3:**
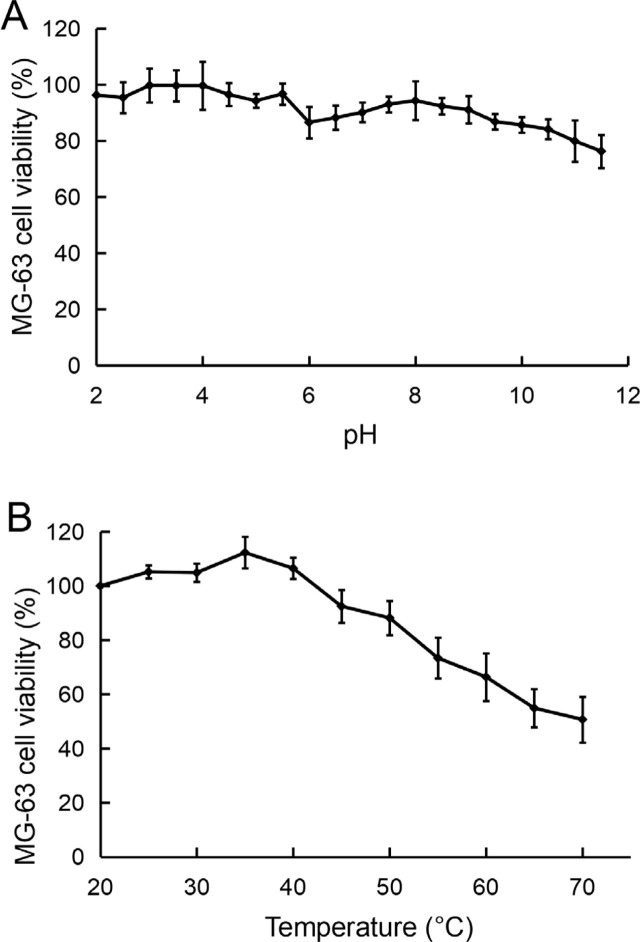
Effect of pH and temperature on the antiproliferative activity of ALAPP. Relative antiproliferative activity of the treated ALAPP against MG-63 osteosarcoma cells was measured using an MTT cell viability assay. **(A)** Effect of pH. ALAPP was incubated at various pH for 60 min at 4 °C. The pH was adjusted to 7.2 before measuring the anti-proliferative activity of the treated ALAPP. **(B)** Effect of temperature. ALAPP was incubated at various temperatures for 60 min followed by cooling in ice bath before measuring the antiproliferative activity of the treated ALAPP. Values are shown as mean (n = 3); error bars indicate standard deviation. ALAPP, antlion antiproliferative polypeptide; MTT, 3-(4,5-dimethylthiazol-2-yl)-2,5-diphenyltetrazolium bromide.

Antiproliferative activity of ALAPP at various pH and temperature was examined using an MTT assay of variously treated cell cultures. The effect of pH on the activity of ALAPP was relatively small. The activity of ALAPP was higher under acidic conditions than it was under alkaline conditions (**Figure 3A**). The activity of ALAPP remained >95% at pH <5.5, and had the highest activity between pH 3 and 4. Although the activity of ALAPP decreased continuously when the pH was >6, >75% of activity remained at pH 11. Like pH, the temperature had a relatively small effect on the anti-proliferative activity of ALAPP (**Figure 3B**). The activity of ALAPP increased from 20 to 40 °C. Although the activity of ALAPP decreased continuously >40 °C, >50% activity remained at 70 °C.

**Figure 4:**
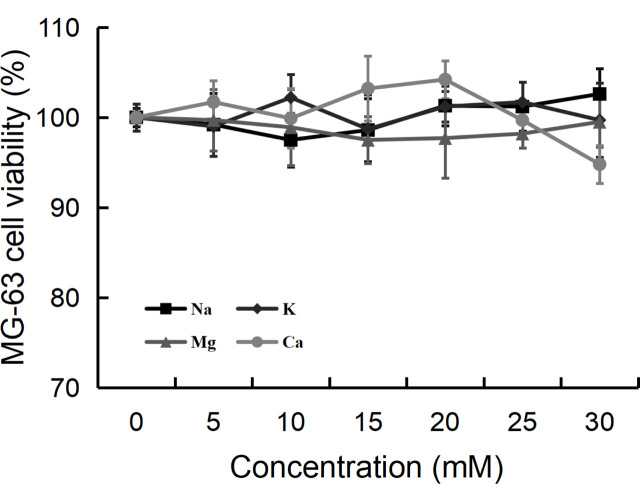
Effect of metal ions on the antiproliferative activity of ALAPP. The mixture of purified ALAPP and metal ion was incubated at 25 °C for 30 min, and the relative antiproliferative activity against MG-63 osteosarcoma cells was measured using an MTT cell viability assay against a control culture without any metal ion. Values are shown as mean (n = 3); error bars indicate standard deviation. ALAPP, antlion antiproliferative polypeptide.

The antiproliferative activity of ALAPP was not sensitive to any of the metal ions tested (**Figure 4**), except that Ca^2+^ showed an inhibitory effect on ALAPP activity at high concentration (>25 mM). The effect of other metal ions on the activity of ALAPP was not significant.

### Antiproliferative activity of ALAPP on osteosarcoma cells

**Figure 5:**
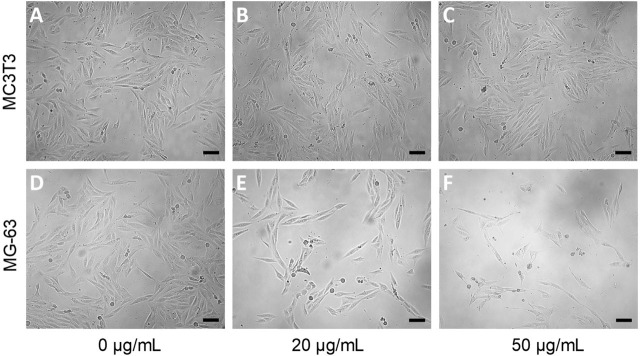
Morphological characteristics and changes in MG-63 osteosarcoma cells and non-neoplastic MC3T3-E1 subclone 14 osteoblast controls effected by ALAPP. Osteosarcoma and osteoblasts were treated with ALAPP at various concentrations or with PBS as a control for 48 h. The morphological characteristics and changes were evaluated using an inverted phase contrast microscope EVOS FL Auto Cell Imaging System 20× LPlan FH objective (catalog No. AMEP-4682; Thermo Fisher Scientific). Untreated cells (**A** and **D**), and the effect of only 2 concentrations, 20 μg/mL ALAPP (**B** and **E**) and 50 μg/mL ALAPP (**C** and **F**), are shown to highlight the substantial differences in cell morphology. **A, B,** and **C**. Mouse osteoblasts MC3T3-E1 subclone 14 derived from osteoblast precursors of the parietal calvaria (ATCC CRL-2594); **D, E,** and **F**. Human osteosarcoma cells MG-63 (ATCC CRL-1427). Scale bars 100 μm. ALAPP, antlion antiproliferative polypeptide; PBS, phosphate-buffered saline.

Morphological characteristics and changes in MG-63 and MC3T3 cells were evaluated after being treated for 48 h by purified ALAPP at a gradient of concentrations (10–50 μg/mL) or with PBS as a control. MC3T3 osteoblasts showed a healthy and intact fusiform morphology at each concentration of ALAPP tested (**Figures 5A–C**). Nevertheless, as their ability to adhere to the well wall was reduced, aging and dying cells no longer formed spindles and contracted to form nearly round cells. However, the MG-63 osteosarcoma cells showed substantial changes in their morphology and features after being treated with ALAPP (**Figures 5D–F**). Their cell size was reduced substantially, and the ability of the 2 polar segments of the spindle to elongate was weakened. At a high concentration of ALAPP (50 μg/mL), the surviving cell morphology became very thin, and the cytoplasm became transparent and granular. The cells, which clumped together in large numbers, had lost their fusiform character.

**Figure 6:**
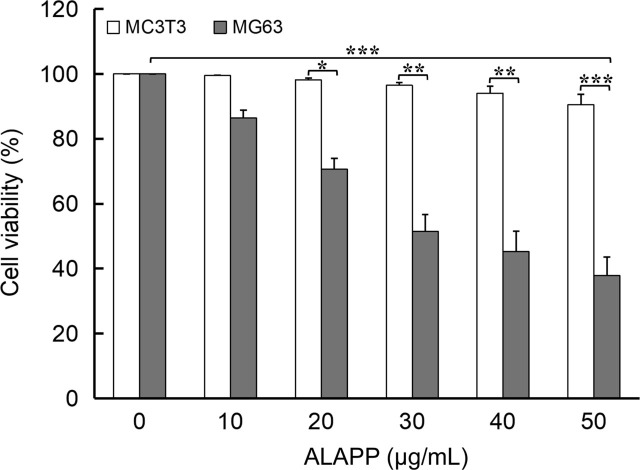
Antiproliferative activity of ALAPP. MG-63 osteosarcoma cells and MC3T3 osteoblasts were treated with ALAPP for 48 h, and antiproliferative activity against the cells was determined using an MTT cell viability assay. Each bar represents the average of n = 3 and error bars indicate the standard deviation. Student *t* tests were conducted to determine differences in antiproliferative activity. ^*^P < 0.05, ^**^P < 0.005, ^***^P < 0.001 vs control. ALAPP, antlion antiproliferative polypeptide.

MG-63 and MC3T3 cell viability after 48 h treatment with ALAPP was determined using an MTT assay. As the concentration of ALAPP increased, the viability of MC3T3 osteoblasts showed a slight decrease (>90%), but MG-63 osteosarcoma cells showed a steady decline in viability (**Figure 6**). When ALAPP reached about 30 μg/mL, the relative viability of MG-63 osteosarcoma was inhibited by 50%. When ALAPP reached 50 μg/mL, the relative viability of MG-63 was only 37.8%. ALAPP had a clear concentration-dependent inhibitory effect on MG-63 osteosarcoma cell viability and, therefore, proliferation.

### Changes in expression of HSF1, HSP90, CDK4, and AKT1 and their mouse homologs

**Figure 7:**
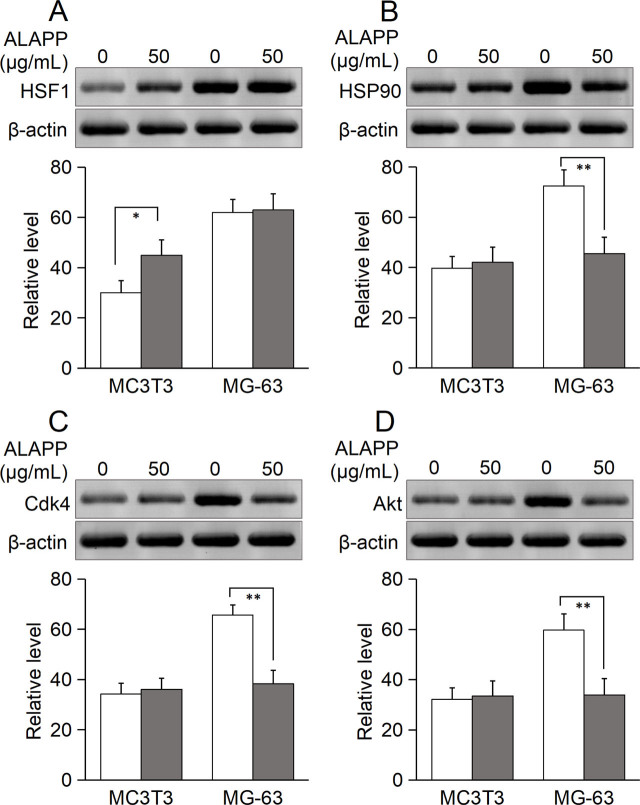
Effect of ALAPP on the levels of HSF1, HSP90, CDK4, and AKT1 expression by MG-63 osteosarcoma cells and level of Hsf1, Hsp90, Cdk4, and Akt1 expression by MC3T3 osteoblasts. The levels of protein expression were determined by western blotting. After treatment with 50 μg/mL ALAPP for 48 h, MC3T3 osteoblasts and MG-63 osteosarcoma cells were collected and mixed with 100 μL xTractor Buffer (Clontech). Total cell protein extracts were adjusted to the same protein concentration using the Bradford method, and their protein components were separated by electrophoresis in a 12% polyacrylamide gel containing SDS and transferred to PVDF membranes. Primary antibodies, each with immunoreactivity for human and mouse homologs of the proteins, were purchased from Sangon Biotech, including the rabbit polyclonal antibodies anti-ACTC1 (catalog No. D224905), anti-HSF1 (catalog No. D220782), anti-HSP90AA1 (catalog No. D220009), anti-CDK4 (catalog No. D120396), and anti-AKT1(Ab-129) (catalog No. D151616). Immunoreactivity was detected with secondary antibody HRP-conjugated goat anti-rabbit IgG (catalog No. D110058, Sangon) using an ECL chemiluminescence substrate (catalog No. T7101A, TaKaRa). Expression of the various proteins was calculated relative to β-actin used as a control. Each bar represents the average of n = 3 and error bars indicate standard deviation. Student *t* tests were conducted to determine differences in expression. ^*^P < 0.05, ^**^P < 0.005 vs untreated control cells. **A**. HSF1. **B.** HSP90. **C.** CDK4, **D.** AKT1. ACTC1, β-actin; AKT1, serine-threonine protein kinase encoded by *AKT1*; ALAPP, antlion antiproliferative polypeptide; CDK4, cyclin-dependent kinase 4; HRP, horseradish peroxidase; HSF1, heat shock transcription factor 1; HSP90, heat shock protein 90 kDa alpha (cytosolic), class A member 1 (HSP90AA1); PVDF, polyvinylidene difluoride.

The peptide sequence of ALAPP has a 56% identity with human heat shock factor binding protein 1 (HSBP1; NCBI sequence ID: NP_001528.1) using UniProt Align (https://www.uniprot.org/align) [17] and 56.8% similarity using the European Molecular Biology Laboratory (EMBL) European Bioinformatics Institute (EBI) Emboss Needle tool (https://www.ebi.ac.uk/Tools/psa/emboss_needle/) [18], and 94% identity (83/85 or 97% positives) with the heat shock factor-binding protein 1 isoform X1 from *Chrysoperla carnea*, a relative of antlion in the order Neuroptera (NCBI sequence ID: XP_044742752.1) using BLAST (https://blast.ncbi.nlm.nih.gov/Blast.cgi) [19]. The human form HSBP1 has an inhibitory activity on a variety of tumors because it binds to HSF1 to inhibit the expression of heat shock proteins (HSPs). Heat shock protein 90 (HSP90) is associated with a variety of signaling molecules, including CDK4 and AKT1, which are widely involved in the occurrence and development of tumors [20, 21]. The levels of expression of HSF1, HSP90, CDK4, and AKT1 and their mouse homologs were analyzed by western blotting. Without ALAPP treatment, the level of HSF1 expression in MG-63 osteosarcoma was about 2-fold the expression of Hsf1 in MC3T3 osteoblasts. After treatment with 50 μg/mL ALAPP, the level of Hsf1 expression was upregulated by about 50% in MC3T3 osteoblasts, but HSF1 expression remained almost unchanged in MG-63 osteosarcoma cells compared with untreated MG-63 cells (**Figure 7A**). HSF1 is a transcriptional regulator of *HSP90*, and positively regulates the expression of HSP90 and other HSPs. Without ALAPP treatment, the HSP90 expression level in MG-63 osteosarcoma was about 82% higher than Hsp90 in MC3T3 osteoblasts. After treatment with 50 μg/mL ALAPP, the level of Hsp90 expression was unchanged in MC3T3 osteoblasts, but compared with the level in untreated cells, HSP90 was downregulated by about 37% in MG-63 osteosarcoma cells (**Figure 7B**). The levels of expression of CDK4 and AKT1, which are client proteins of HSP90, are associated with HSP90. Without ALAPP treatment, the levels of CDK4 and AKT1 expression in MG-63 osteosarcoma cells were about 92.1% and 86% higher, respectively, than those of Cdk4 and Akt1 expression in MC3T3 osteoblasts. After treatment with 50 μg/mL ALAPP, the levels of expression of Cdk4 and Akt1 were unchanged in MC3T3 osteoblasts, but compared with levels in untreated cells, the levels of CDK4 and AKT1 expression were downregulated by about 42% and 43%, respectively, in MG-63 osteosarcoma cells (**Figures 7C** and **7D**). Overall, in MC3T3 osteoblasts, ALAPP upregulated Hsf1, but failed to upregulate Hsp90, Cdk4, and Akt1. However, in MG-63 osteosarcoma cells, ALAPP failed to upregulate HSF1, and HSP90, CDK4, and AKT1 were downregulated to about the level seen in osteoblasts. These findings suggest that the levels of HSP90, CDK4, and AKT1 expression are comparable to those of their homologs in osteoblasts, but could not maintain the survival of the osteosarcoma cells.

## Discussion

An 85-amino-acid polypeptide with a calculated molecular weight of about 9.7 kDa and remarkable antiproliferative activity against MG-63 osteosarcoma cells was purified from antlion, and identified as a homolog of HSBP1.

A point emphasized in TCM descriptions of formulae containing antlion recommended for treating tumors is that the antlion powder should be dissolved in warm water [10], although boiling water is used for most formulae in TCM. The active ingredients in TCM that cannot be continuously boiled in water are usually not resistant to high temperatures. Naturally occurring small molecules are usually the active components of botanically based TCM, while the active components of animal substance-based TCM are often peptides. Therefore, we screened the peptides found in antlion for their antiproliferative activity against osteosarcoma cells. Gel filtration chromatography indicated 2 peaks (Peak 1 and Peak 6) of antiproliferative activity. Peak 1 would mainly consist of proteins >100 kDa. The active component with inhibitory activity against osteosarcoma in Peak 1 is perhaps the 165 kDa protein toxin, whose paralytic activity against cockroaches was identified by others as about 130-fold higher than that of tetrodotoxin [22, 23]. Because large proteins have low stability and poor cell permeability compared with smaller polypeptides, Peak 6, comprised of low-molecular-weight polypeptides <10 kDa, was selected for further examination.

Microbial communities, which are symbiotic in the esophagus and intestines of insects, often play important roles in the nutrition, reproduction, development, and even behavior of their hosts. A wide variety of bacteria have been reported to be endosymbiotic in the gut and esophagus of antlion [6, 24, 25]. These endosymbiotic bacteria of antlion play important roles in their hunting behavior, because some toxins, which have been implicated as contributing to the paralyzation and death of prey, are proven to be produced by bacterial isolates. Yoshida et al. [26] reported an insecticidal 63 kDa protein purified from the saliva of antlion, as produced by the endosymbiont *Enterobacter aerogenes*. The protein was characterized as a GroEL homolog, acting as a chaperonin to ensure correct folding and assembly of proteins under heat shock or other stress [27]. Antlions can avoid the toxicity of these proteins produced by endosymbiotic bacteria. However, the molecular mechanism by which antlions use these bacterial proteins to kill their prey without harming themselves is not yet known. Perhaps the mechanism is related to the interdependence of antlion and bacteria, which has evolved over millions of years of symbiosis. If the toxin produced by endosymbiotic bacteria of antlions is a GroEL homolog of the HSP60 family of chaperonins, ALAPP may be a type of polypeptide acting on the HSP pathway. Indeed, the molecular weight and sequence of ALAPP are homologous to those of HSBP1, which has inhibitory activity on a variety of tumors because of its binding to HSF1 to inhibit the expression of HSPs [28]. Whereas, GroEL is the major HSP of *Escherichia coli* and belongs to the chaperonin HSP60 family. While HSPs prevent misfolding of proteins and promote the refolding and proper assembly of unfolded polypeptides generated under conditions of thermal and other stress, the transcriptome of HSF1 and HSPs regulates multiple traits associated with malignant transformation and tumorigenesis [29].

Not only is ALAPP homologous to heat shock factor-binding protein 1 isoform X1 from *Chrysoperla carnea* (a relative of antlion in the order Neuroptera), but the amino acid sequence of ALAPP has about 57% similarity with human HSBP1. Therefore, we speculate that ALAPP inhibits the expression of HSPs by inhibiting the activity of HSF1, thus inhibiting osteosarcoma cell proliferation. However, another question is how normal cells evade the antiproliferative activity of ALAPP. Analysis of the expression of a variety of related protein factors by western blotting (**Figure 7**) indicated that the growth of MC3T3 osteoblasts was not inhibited by ALAPP at <50 μg/mL, upregulating Hsf1 to maintain Hsp90, Cdk4, and Akt1 in a relatively constant range. However, MG-63 osteosarcoma had preemptively activated HSF1 expression (by about 2-fold the expression of osteoblasts) to maintain normal survival of the osteosarcoma cells. When the activity of HSF1 was substantially inhibited by ALAPP, the osteosarcoma failed to express more HSF1, presumably due to the depleted potential expression pool. As the concentration of ALAPP was increased, the expression of Hsf1 in osteoblasts continued to rise until it reached the level of its homolog in the osteosarcoma, and then the proliferation of osteoblasts began to be gradually inhibited. These findings suggest that HSF1 has a controllable plastic expression pattern in various cells, but that the level of its expression in cancer cells is at its limit.

If ALAPP is a protein factor of the HSBP1 class, then the biochemical properties of ALAPP are well understood. The HSBP1 family members are comprised solely of a putative coiled-coil oligomerization domain without any other readily recognizable structural or functional motif [30]. In the α-helix of HSBP1, all of residues are hydrophobic with an exception of Ser31, as it is energetically unfavorable to the structure stability, but likely bears some biological function [30, 31]. If the mode of action of ALAPP is to interact with the DNA-binding protein HSF1, as shown for HSBP1 located in the nucleus [32], it remains unclear how ALAPP incubated with cells can cross the cellular membrane and reach the nucleus when it apparently has an anionic amphipathic structure with a pI 4.11. Nevertheless, ALAPP has high stability to a wide range of temperature and pH, which makes it an ideal peptide-based drug, and reflects the advantages of peptide-based agents. ALAPP showed its strongest antiproliferative activity against the MG-63 osteosarcoma at lower pH (pH 3–4), suggesting that ALAPP could be developed as an oral drug with resistance to gastric acidity. The stability of the peptide warrants further investigation by techniques to investigate secondary structure, such as circular dichromism. However, in the present study we were limited by the low yield of the purified polypeptide.

The present study is limited in that we did not use HSBP1 or any other positive control. Nevertheless, the purified polypeptide was used for all cell viability and protein expression experiments. A limitation of many TCM is that only efficacy is known, but the specific substance in the mixtures that produce the effects is unknown. The substance that produces the effect may be one, or a combination or synergistic effect of the many substances in the mixture. Thus, the antitumor effect of TCM formulae containing antlion may also be attributable to another substance such as polysaccharides [8, 9], mercury [10], flavonoids [33], or cantharids [10].

The MC3T3-E1 subclone 14 cell line can exhibit high levels of osteoblast differentiation and is a good model for studying osteoblast differentiation in vitro, having behavior similar to primary calvarial osteoblasts, but it is a spontaneously transformed (immortalized) mouse cell line. As such, caution should be used when extrapolating these results to normal cells, and even more, to normal human cells.

## Conclusion

ALAPP purified from antlion is an 85-aminoacid 9.7 kDa polypeptide with 56% sequence identity with HSBP1. ALAPP has significant antiproliferative activity against MG-63 osteosarcoma cells, but not non-neoplastic MC3T3-E1 osteoblasts. We speculate that non-neoplastic cells may evade the antiproliferative effect of ALAPP by upregulating HSF1 to maintain their HSP90, CDK4, and AKT1 expression at a relatively constant level.
